# Gestational diabetes as a risk factor for GBS maternal rectovaginal colonization: a systematic review and meta-analysis

**DOI:** 10.1186/s12884-024-06694-7

**Published:** 2024-07-20

**Authors:** Vicki Mercado-Evans, Jacob J. Zulk, Zainab A. Hameed, Kathryn A. Patras

**Affiliations:** 1 Department of Molecular Virology and Microbiology, Baylor College of Medicine, One Baylor Plaza, MS 385, Houston, TX 77030 USA; 2 Medical Scientist Training Program, Baylor College of Medicine, Houston, TX 77030 USA; 3 Alkek Center for Metagenomics and Microbiome Research, Baylor College of Medicine, Houston, TX 77030 USA

**Keywords:** Group B *Streptococcus*, *Streptococcus agalactiae*, Gestational diabetes, Diabetes mellitus, Type 1 diabetes, Type 2 diabetes, Vaginal colonization, Neonatal outcomes, Vaginal microbiome

## Abstract

**Background:**

Maternal rectovaginal colonization by group B *Streptococcus* (GBS) increases the risk of perinatal GBS disease that can lead to death or long-term neurological impairment. Factors that increase the risk of rectovaginal GBS carriage are incompletely understood resulting in missed opportunities for detecting GBS in risk-based clinical approaches. There is a lacking consensus on whether gestational diabetes mellitus (GDM) is a risk factor for rectovaginal GBS. This systematic review and meta-analysis aims to address current conflicting findings and determine whether GDM should be clinically considered as a risk factor for maternal GBS colonization.

**Methods:**

Peer-reviewed studies that provided GDM prevalence and documented GBS vaginal and/or rectal colonization in women with and without GDM were included in this analysis. From study inception to October 30, 2023, we identified 6,275 relevant studies from EMBASE and PUBMED of which 19 were eligible for inclusion. Eligible studies were analyzed and thoroughly assessed for risk of bias with a modified Newcastle-Ottawa Scale that interrogated representativeness and comparability of cohorts, quality of reporting for GDM and GBS status, and potential bias from other metabolic diseases. Results were synthesized using STATA 18 and analyzed using random-effects meta-analyses.

**Results:**

Studies encompassed 266,706 women from 10 different countries, with study periods spanning from 1981 to 2020. Meta-analysis revealed that gestational diabetes is associated with a 16% increased risk of rectovaginal GBS carriage (OR 1.16, CI 1.07–1.26, *P* = 0.003). We also performed subgroup analyses to assess independent effects of pregestational vs. gestational diabetes on risk of maternal GBS carriage. Pregestational diabetes (Type 1 or Type 2 diabetes mellitus) was also associated with an increased risk of 76% (pooled OR 1.76, CI 1.27–2.45, *P* = 0.0008).

**Conclusions:**

This study achieved a consensus among previously discrepant observations and demonstrated that gestational diabetes and pregestational diabetes are significant risk factors for maternal rectovaginal carriage of GBS. Recognition of GDM as a risk factor during clinical decisions about GBS screening and intrapartum antibiotic prophylaxis may decrease the global burden of GBS on maternal-perinatal health.

**Supplementary Information:**

The online version contains supplementary material available at 10.1186/s12884-024-06694-7.

## Introduction

Group B *Streptococcus* (GBS) remains a leading cause of neonatal morbidity and mortality across the globe despite nearly two decades of systematic implementation of preventative approaches that include universal maternal screening or risk-based administration of antibiotics during delivery [1]. GBS colonizes the vaginal and/or gastrointestinal tract of about 18% of pregnant women [2, 3]. Neonates can acquire GBS during passage through the vaginal canal during delivery, and GBS also has the capacity to cause ascending intraamniotic infection. While some women and their neonates are colonized without symptoms, GBS can cause devastating complications and disease such as spontaneous abortion, preterm labor, stillbirth, and neonatal sepsis and meningitis [1, 4]. The mechanisms driving the divergence in GBS pathogenic vs. commensal behavior is poorly understood. Currently, the following maternal factors are clinically recognized for increasing the risk of GBS neonatal disease and are used to identify women who should be given intrapartum antibiotic prophylaxis (IAP): (1) previous infant with early-onset GBS disease (EOGBS), (2) GBS bacteriuria during the current pregnancy, (3) temperature > 38 °C during labor, (4) prolonged rupture of membranes (PROM) > 18 h, or (5) delivery at < 37 weeks of gestation [5]. Some countries practice this risk-based approach and others implement universal screening of women around 35 weeks of gestation, with subsequent IAP for those who have rectovaginal GBS carriage. Screening based approaches are associated with enhanced protection against neonatal GBS early onset disease (EOD; occurring in the first week of life) compared to risk-based strategies [5], which suggests that we have yet to understand all of the maternal factors that predict GBS disease. Additionally, up to 46% of cases of EOD occur in the absence of the risk factors currently used for clinical decision making [5–7].

One possible additional risk factor for maternal rectovaginal GBS colonization is gestational diabetes mellitus (GDM) which affects approximately 14% of pregnancies worldwide [8]. GDM, diabetes that develops during pregnancy, is a state of heightened insulin resistance, insufficient pancreatic insulin production, hyperglycemia, immune dysregulation, and altered vaginal microbial composition [9–12]. This systemic disruption to maternal physiology leads to an increased risk of complications including preterm birth, pre-eclampsia, and a long-term increased risk of cardiovascular disease in both women and their children. In clinical cohort studies, infants born to women with gestational diabetes are at greater risk of early onset culture-verified GBS sepsis [13], late onset clinical sepsis [14] and extended hospital stay [15]. Considering that rectovaginal GBS carriage is the primary risk factor for GBS neonatal sepsis, women with GDM may have greater GBS colonization rates thereby imparting increased risk of neonatal disease. Observational clinical studies have reported conflicting findings on the association between diabetes (pregestational Type 1 or Type 2 and GDM) and GBS carriage; some have found increased risk of GBS colonization [16–21] in diabetic pregnant women (pregestational and/or gestational), while others found no association [15, 22, 23]. Several of these studies did not specifically distinguish pregestational (Type 1 or Type 2 DM) from GDM. Although these metabolic diseases share several features, the acuity and specificity to pregnancy of GDM lends unique insight into the pathogenic potential of group B *Streptococcus*.

The aim of this study was to conduct a systematic review and meta-analysis of the risk for rectovaginal GBS carriage in women affected by GDM. Resolving whether GDM is an independent risk factor for maternal GBS colonization is essential for closing the gap in current IAP approaches; a critical step towards reducing the global burden of GBS-associated neonatal morbidity and mortality.

## Materials and methods

### Search strategy and study selection

**Fig. 1:**
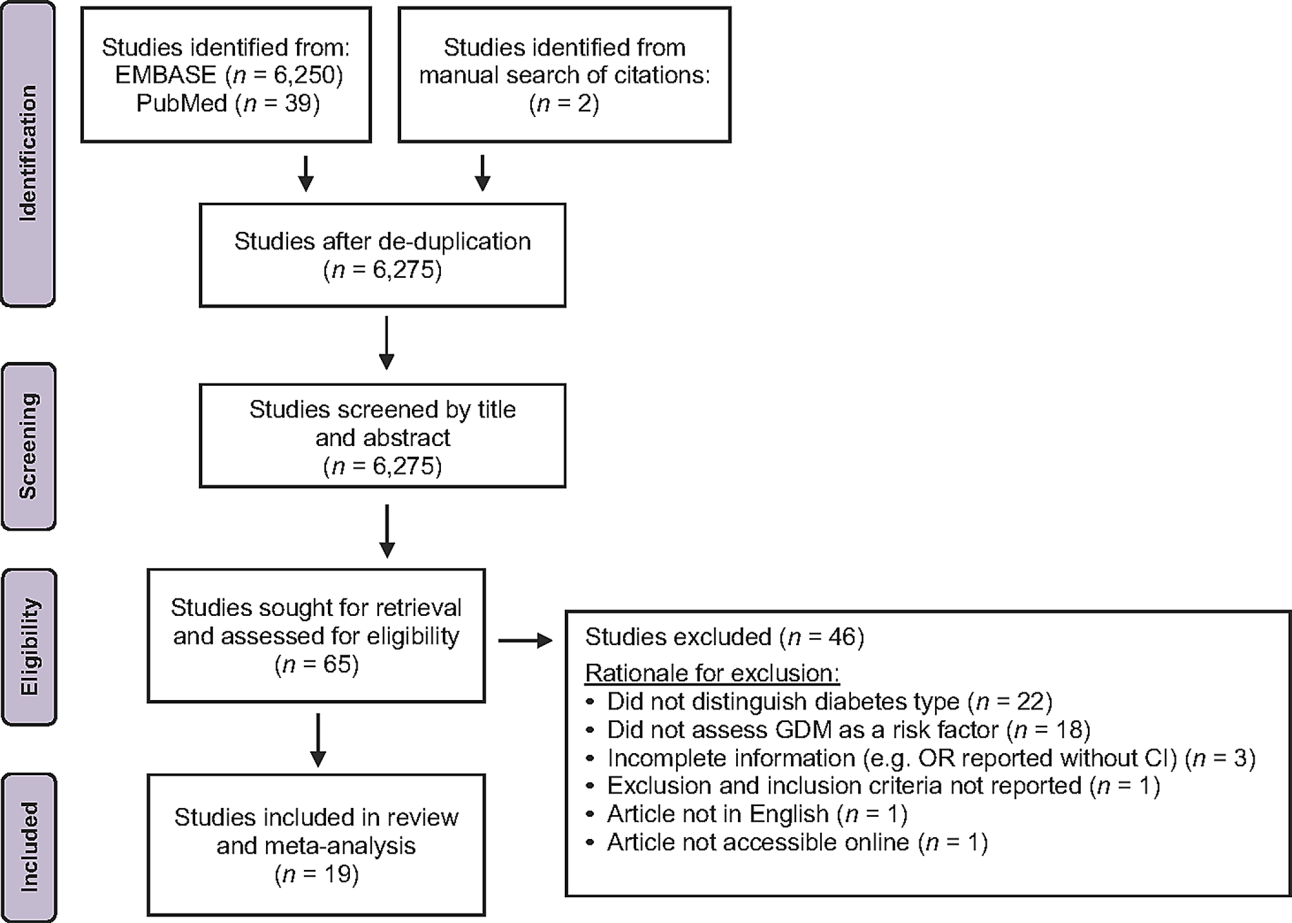
Study identification, screening, and selection process. Flow diagram of selection of the included studies

Studies were identified through a database search that included PubMed and EMBASE, which encompassed MEDLINE and preprints as sources (Fig. 1). The search strategy implemented search terms intended to capture two kinds of studies: (1) Those that specifically assessed GBS maternal colonization and/or neonatal transmission in women with gestational diabetes, and (2) studies on GBS maternal colonization prevalence which included information about gestational diabetic status and respective GBS status.

As such, a combination of the following search terms was implemented: gestational diabetes or GDM, chorioamnionitis, newborn and sepsis or cocci and sterile site, and group b streptococcus or GBS or streptococcus agalactiae, English, humans. The following search terms were used as filters followed by the word not: in vitro, ex vivo, animal, tilapia, zebra fish, bovine, breast milk, phylogeny, case study, cells, case report, urinary tract infection, non-pregnancy, endocarditis, murine, mouse, primate. Reviews, conference abstracts and editorials were excluded from our search. The literature search was restricted to human studies published in English, with no study period restrictions (Supplemental Table 1). The last query was performed on October 30, 2023. Titles and abstracts were screened for adherence to the inclusion and exclusion criteria detailed below. We did not prospectively prepare or register the protocol and study, but we adhered to the Preferred Reporting Items for Systematic Reviews and Meta-Analyses (PRISMA) for this systematic review and meta-analysis.

### Inclusion and exclusion criteria

The inclusion criteria for this study were: peer-reviewed studies that documented GBS vaginal and/or rectal colonization in expectant mothers, with information about the proportion of women who were clinically diagnosed with GDM. Studies were required to provide the proportion of women who did not have GDM. We included studies that potentially had women with pregestational diabetes in the control, non-GDM, group but we stratified analyses accordingly. Reporting of GDM diagnosis and maternal GBS vaginal and/or rectal colonization was accepted through medical records or diagnosis from medical professionals. Studies were included irrespective of sample type used to determine GBS colonization (vaginal, rectal, or perianal region) and studies employing molecular or biochemical detection of GBS were included. Studies with self-collected vaginal swab samples (*n* = 1) were included. Additionally, observational, baseline data from interventional, case-control, retrospective and prospective, cross sectional and cohort studies were all included. The exclusion criteria for this study were: case studies, reviews, or letters to the editor, lack of a GBS negative or non-GDM control group, published in a language other than English, studies that did not explicitly state diabetes type (GDM vs. pregestational diabetes: Type 1 or Type 2 DM), or missing critical information such as exclusion/inclusion criteria or GBS and/or GDM prevalence in the study population. For relatively contemporary studies (published in the past decade) that did not specify diabetes type, we emailed corresponding authors to acquire information about the number of participants that had GDM vs. Type 1 vs. Type 2 diabetes and their GBS status (*n* = 9 studies). One reviewer screened titles and abstracts, and three reviewers independently screened full texts to assess studies for eligibility. Reasons for exclusion of each eligible study are provided (Supplemental Table 2).

### Data extraction and assessment of quality and bias

Two independent reviewers extracted data that included 29 variables: Author, year, PMID, study period, country, screening IAP criteria during time of study, facility, study design inclusion criteria, exclusion criteria, total number of pregnancies/deliveries assessed, number of women with GDM, number of patients with Type I or Type II DM, number of non-diabetic controls, number of women who were GBS positive, number of women who were GBS negative in the non-diabetic, pregestational diabetic and GDM groups, reported OR/RR of GBS colonization in women with GDM, additional relevant reported findings (i.e. chi-square, ORs for nondiabetic vs. all diabetic women), mean age of women, GDM diagnostic criteria and ascertainment, information about medical management of GDM, gestational age at time of GBS screen, GBS detection method (culture, PCR, vaginal, rectal etc.), findings on maternal GBS invasive disease, findings on neonatal GBS infection and potential confounders.

All eligible studies were assessed for quality and risk of bias by two independent reviewers via an adapted Newcastle-Ottawa Scale [24] (Supplemental Table 3) which focused on four broad criteria: (1) how representative the groups were of the greater communities from which the study was conducted, (2) comparability of the groups to each other with respect to various characteristics (maternal age, BMI, racial/ethnic representation, socioeconomic status, etc.), (3) quality of outcome assessment (ascertainment of GBS and GDM status), and (4) potential of bias from other metabolic diseases in each group such as obesity and pregestational diabetes (in non-GDM control group). Each assessment category was scored, and the sum was used to determine overall quality and risk of bias for each study. Any discrepancies greater than 2 points for any category were resolved via a discussion to achieve consensus. A total score $\:\le\:$ 3 was considered low bias,$\:>$ 3 and$\:\:\le\:$ 6 indicated moderate bias, and $\:\ge\:$ 6 was classified as high risk of bias. The certainty of evidence was graded into four levels (very low, low, moderate, high) based on GRADE [25] guidelines and this was used to assess the overall quality of evidence.

### Data synthesis and analysis

Unadjusted ORs were calculated for studies in which only prevalence data were provided, otherwise reported ORs for GDM or pregestational diabetes were utilized. OR calculation and analysis were performed using STATA 18. To calculate ORs, we compared the odds of rectovaginal GBS carriage in women with GDM versus women without GDM. For the sub-analysis of women with pregestational diabetes, we compared the odds of rectovaginal GBS carriage to nondiabetic women, or to women with GDM. For sub-analysis of pregestational diabetes, Piper et al., 1999 [15] could not be included because this study accounted for effects of pregestational diabetes by excluding this population entirely from their study. Forest plots display prevalence, individual ORs and 95% confidence intervals (CI) and meta-analysis of pooled ORs with random effects modeling. The I^2^ index was used to assess the impact of study heterogeneity on study estimate variance [24], with low, moderate and high heterogeneity indicated by I^2^ of 25%, 50% and 75% respectively [26]. Sensitivity analysis included exclusion of studies that did not document or control for pregestational diabetic prevalence and exclusion of studies with high risk of bias. The remaining 8 studies then underwent a meta-analysis of pooled ORs with random effects modeling. Publication bias was assessed with funnel plots of the ORs (natural logarithm) against the inverse of the standard error and Egger’s regression test, with *P* < 0.05 indicating a significantly asymmetric funnel plot and thus significant publication bias.

## Results

### Study characteristics

The initial search identified 6,275 studies, of which 65 articles passed screening and were subjected to full-text assessment for eligibility (Fig. 1). 19 articles with study periods spanning from 1981 to 2020 were eligible based on the aforementioned criteria, with exclusion of 46 studies for various reasons (Supplemental Table 2). Table 1 provides a summary of study characteristics. The total number of women included in this systematic review and meta-analysis is 266,706; there were 18,715 women with GDM, 2,598 with pregestational diabetes, and 195,545 without GDM. The studied populations are representative of many communities across the globe with inclusion of Australia, Brazil, China, Finland, Lebanon, Mexico, Morocco, Nigeria, Spain, and the United States. Of these countries, none were of low-income, three were of lower-middle income, three were of upper-middle income, and four were of high-income as determined by the 2023 World Bank guidelines [27]. 6/19 were multicenter studies [14–16, 28–30] and routine screening for GBS colonization and administration guidelines for intrapartum antibiotic prophylaxis (IAP) was not an established practice at the time for 52% of studies (Table 2). Studies also consisted of a mix of prospective (53%) [14, 15, 17, 18, 29, 31–35], retrospective (16%) [16, 36, 37], cross-sectional (5%) [30], case-control (16%) [28, 38, 39], and population-based cohort (11%) [36, 40] study designs. Rectovaginal, vaginal and/or perineal GBS carriage was determined by culture for all studies: 14 studies performed rectovaginal sampling [14, 15, 17, 18, 29, 31–35, 37, 39–41], 3 solely assessed vaginal carriage [30, 36, 38], and 2 studies used diagnostic codes from hospital records and thus sampling method cannot be specified [16, 28]. 5 studies [15, 31, 33, 37, 41] performed culturing and molecular or biochemical identification as recommended by CDC guidelines whereas the remaining 14 studies had methods incongruent with guidelines or did not provide enough detail. 6 were found to have low risk of bias, 8 had moderate risk of bias, and 5 had high risk of bias (Table 1 and Supplemental Table 4).

**Table 1:** Characteristics of studies included in this systematic review of the association between gestational diabetes and rectovaginal GBS colonization

Author, year	Study period	Country	Study Design	Inclusion (I) & Exclusion (E) Criteria	Mean age (SD)	GDM diagnostic criteria and ascertainment	GBS detection method	Potential confounders	Risk of bias
Matorras, 1988 [17]	1981–1985	Spain	Prospective	Women randomly selected. No criteria specified.	28.0 (6.1)	Oral GTT around 20 w. Coustan & Lewis Criteria. Pregestational diabetes based on history of diabetes or fasting glycemia > 140 mg/100mL.	Rectal and vaginal swabs followed by culture detection.	Of the 1,050 patients, 729 had complications that were not specified.	High
Raimer, 1997 [27]	11 months but year is not reported	USA	Case-control	I: All pregnant women presenting to the clinic. E: HIV+, history of substance abuse, current STD.	Diabetic 30.6 (6.2) non-diabetic 28.5 (6.8) (*P* = 0.02)	Oral GTT 24–28 w, considered normal below 140 mg/dL, abnormal screen (50 g challenge) was followed by 3 h GTT (100 g), two elevated values considered abnormal.	Vaginal swab followed by culture detection.	Significant difference in maternal age between diabetic and control groups.	Moderate
Ramos, 1997 [18]	January 1995-March 1996	USA	Prospective	I: Singleton gestation, intact membranes at enrollment, otherwise uncomplicated pregnancy. E: HIV+, chronic steroid therapy, cervical incompetence, multifetal gestation.	Diabetic 27.0 (6.5) non-diabetic 24.6+/- (6.4) (*P* = 0.02)	Oral GTT 24–28 w. At least 2 abnormal readings: fasting glucose greater than or equal to 105 mg/dL, 1 h glucose (50 g challenge) greater than or equal to 190 mg/dL, 2 h glucose greater than or equal to 165 mg/dL, 3 h glucose greater than or equal to 145 mg/dL.	Rectal and vaginal swabs followed by culture detection.	N/A. Regression analyses controlled for maternal age, race, and obesity.	Low
Piper et al., 1999 [15]	January 1992-June 1994 (diabetic cohort); April 1992-December 1992 (nondiabetic cohort)	USA	Prospective	E: Women with previously affected infants and/or pregestational diabetes.	GDM 28.5 (6.2) Non-diabetic 23.6 (5.5) (*P* < 0.05)	Abnormal glucose tolerance test with universal screening.	Rectovaginal swab followed by culture detection.	Diabetic cohort was significantly older, of higher parity, and less likely to deliver vaginally compared to nondiabetic controls.	High
Stapleton et al., 2005 [28]	1997–2002	USA	Case-control	I: All singleton gestation births in Washington State. E: Patients with missing data.	Cases 27.6 (6.1) Controls 27.4 (6.2)	Data extracted from hospital records.	ICD codes for confirmed GBS maternal colonization or suspected carrier.	As acknowledged by authors, risk of disease misclassification and cannot distinguish women who were truly GBS negative vs. those who were not screened.	Low
Medugu et al., 2017 [14]	May-September 2014	Nigeria	Prospective	I: Third trimester. E: Multifetal gestation, placenta previa, or elective caesarean section.	29.8 (5)	Interviews, questionnaires, and hospital records.	Vaginal and rectal swabs and culture detection with confirmation via PathoDxtra Strep Grouping kit.	Prevalence and effects of comorbidities were not considered.	Moderate
Chen et al., 2018 [29]	January-June 2017	Western China	Prospective	I: >35 w gestation routine prenatal care or at the time of delivery. E: Multifetal gestation, GBS culture results not available.	Not reported.	Data extracted from hospital records.	Vaginal and rectal swabs and culture and biochemical detection.	Prevalence of pregestational diabetes not reported, nor other indicators of metabolic stress (BMI).	Low
Moraleda et al., 2018 [30]	March-July 2013	Morocco	Prospective	I: 35–37 w who attended general or high-risk prenatal visits. High risk included pre-existing chronic conditions, complications in previous pregnancies, or maternal, fetal, or placental risks in current pregnancy, or women enrolled at time of delivery without membrane rupture or hemorrhage. No exclusion criteria described.	27 +/- 6.15	Demographic, socio-economic, and clinical data collected through standardized questionnaires.	Recto-vaginal swabs and culture detection.	Control group might contain women with pregestational diabetes as this was not mentioned in exclusion criteria and prevalence of pregestational diabetes in GBS carriers and non-carriers was not described.	High
Dai et al., 2019 [31]	Not reported	China	Prospective	I: Native (Chinese), 20–46 years old, 35–37 w, no sexual intercourse or antibiotic application within recent 3 months. No exclusion criteria described.	GBS+ 30.04 (3.22) GBS- 30.67 (3.51)	Not reported.	Recto-vaginal swabbing followed by PCR on extracted DNA within 24 h of collection.	Very strict inclusion criteria and did not assess prevalence of pregestational diabetes, nor differences in BMI.	High
Edwards et al., 2019 [16]	January 1, 2003 - December 31, 2015	USA	Retrospective cohort	All pregnant women during the timeframe were eligible. No exclusions.	GBS+ 28.0 (6.2) GBS- 28.7 (6.2)	ICD codes from hospital records.	Used diagnostic codes from any time during pregnancy to determine positivity.		Low
Furfaro et al., 2019 [32]	2015–2017	Australia	Prospective cohort	I: 16 + years old, Nulliparous/multiparous, Gestational age of less than 22 w at enrollment, understand, read, and speak English. E: Highly dependent on medical care, cognitive impairment/intellectual disability, illegal drug use, antibiotic/antifungal use within 2 weeks of sample collection, multiple pregnancy (twins, etc.).	32 (16–50)	Not reported.	Self-recto-vaginal swabbing with two swabs at each site. One swab used for multiplex PCR and the other for culture detection.		Low
Ji et al., 2019 [33]	January 2016 - December 2016	China	Population-based cohort	I: All pregnant women were screened at 35–37 w, but prior to 35 w were also tested if delivery occurred before then. E: Women whose pregnancy did not result in labor.	Not provided.	Hospital records.	Recto-vaginal swab performed by physician followed by RT-PCR and culture detection.		Moderate
Manzanares et al., 2019 [34]	2012–2014	Spain	Case-Control	I: Delivery of a single live fetus after 26 w, BMI and GBS culture results available. E: Stillbirth, or less than 26 w.	GBS+ 30.84 (5.8) GBS- 30.61 (5.66)	Not reported.	Positive culture from a recto-vaginal swab at 35–37 w or GBS bacteriuria any time during pregnancy.	Urine screening replaced culture if positive any time during pregnancy, no description of culture methods.	Moderate
Zhu et al., 2019 [35]	April 1, 2014 - March 31, 2017	China	Retrospective cohort (population based)	I: Pregnant women 35–37 w of gestation or with preterm delivery who submitted vaginal swabs. E: Women who did not undergo GBS screening, prenatal diagnosis of fetal malformation, greater or equal to three prior abortions, antibiotic usage in the week prior to admission.	GBS+ 29.7 (4.30) GBS- 29.51 (4.32)	Questionnaire.	Vaginal swabs followed by culture on chromogenic agar. Neonates were screened by tracheal secretion, gastric fluid, and blood sample culture.	Rectal swabs were not collected, no PCR was performed.	Moderate
Alfouzan et al., 2021 [36]	Not reported	Lebanon	Prospective cross-sectional	Not reported.	Not reported.	Questionnaire utilized to gather sociodemographic and clinical information.	Vaginal swabs followed by culture detection.	Control group might include women with pregestational diabetes. Only vaginal swabs were collected which may underestimate GBS colonization.	Moderate
Huang et al., 2021 [37]	June 2019- December 2020	China	Prospective	I: No vaginal GBS colonization before pregnancy, single gestation, viable fetus, no antibiotic use during pregnancy, no sexual activity for 3 days preceding sample collection and no vaginally administered drugs or vaginal lavages 2 weeks before sample collection. E: Malignant tumor, infectious diseases, comorbidities involving heart, liver lungs and other organs, genital tract malformation or incomplete medical records.	Not reported.	Determined from medical records.	Vaginal and rectal swabs followed by PCR detection.	Control group might include women with pregestational diabetes.	Moderate
Place et al., 2021 [38]	January 2014-December 2017	Finland	Retrospective	I: Women undergoing labor induction, singleton gestation, cephalic presentation, unfavorable cervix, intact amniotic membranes. E: Women for which GBS testing was indeterminant.	31.4 (5.4)	2 h 75 g oral glucose tolerance test.	Vaginal and rectal swabs PCR detection (Xpert GBS).	All women in this study had an unfavorable cervix.	Moderate
Del Carmen Palacios-Saucedo, et al., 2022 [39]	April 2017 - December 2018	Mexico	Prospective	Not reported.	Median and range of GBS colonized: 25 (19–37), median and range of non-colonized: 27 (14–43)	Not reported.	Recto-vaginal swabbing followed by culture and biochemical identification.	No definition of GDM.	High
McCoy et al., 2023 [40]	December 2013- February 2017	USA	Secondary analysis of prospective cohort study	I: singleton pregnancy and presented prior to 20 w. E: Major fetal anomaly, HIV positive, history of organ transplant, chronic steroid use.	28.6 (+/- 6.3)	Not reported.	Recto-vaginal swabs followed by culture detection per CDC guidelines	GDM diagnostic criteria and differences in severity of disease may impact findings.	Low

**Table 2:** GBS screening and management practices for clinical sites by study

No established GBS screening or IAP guidelines at the time of study	Risk based screening and IAP for positive screens	Universal screening and IAP for positive screens	Screening and IAP guidelines not specified in study
Matorras et al., [17] Ramos et al., [18] Piper et al., [15] Medugu et al., [14] Chen et al., [20] Dai et al., [32] Zhu et al., [36] Huang et al., [34] Place et al., [37] Del Carmen Palacios-Saucedo, et al., [35]	Raimer et al., [38] Moraleda et al., [29]	Stapleton et al., [28]; Edwards et al., [16] Furfaro et al., [33] Ji et al., [40] McCoy et al., [41]	Manzanares et al., [39] Alfouzan et al., [30]

### Association between gestational diabetes and maternal GBS colonization

**Fig. 2:**
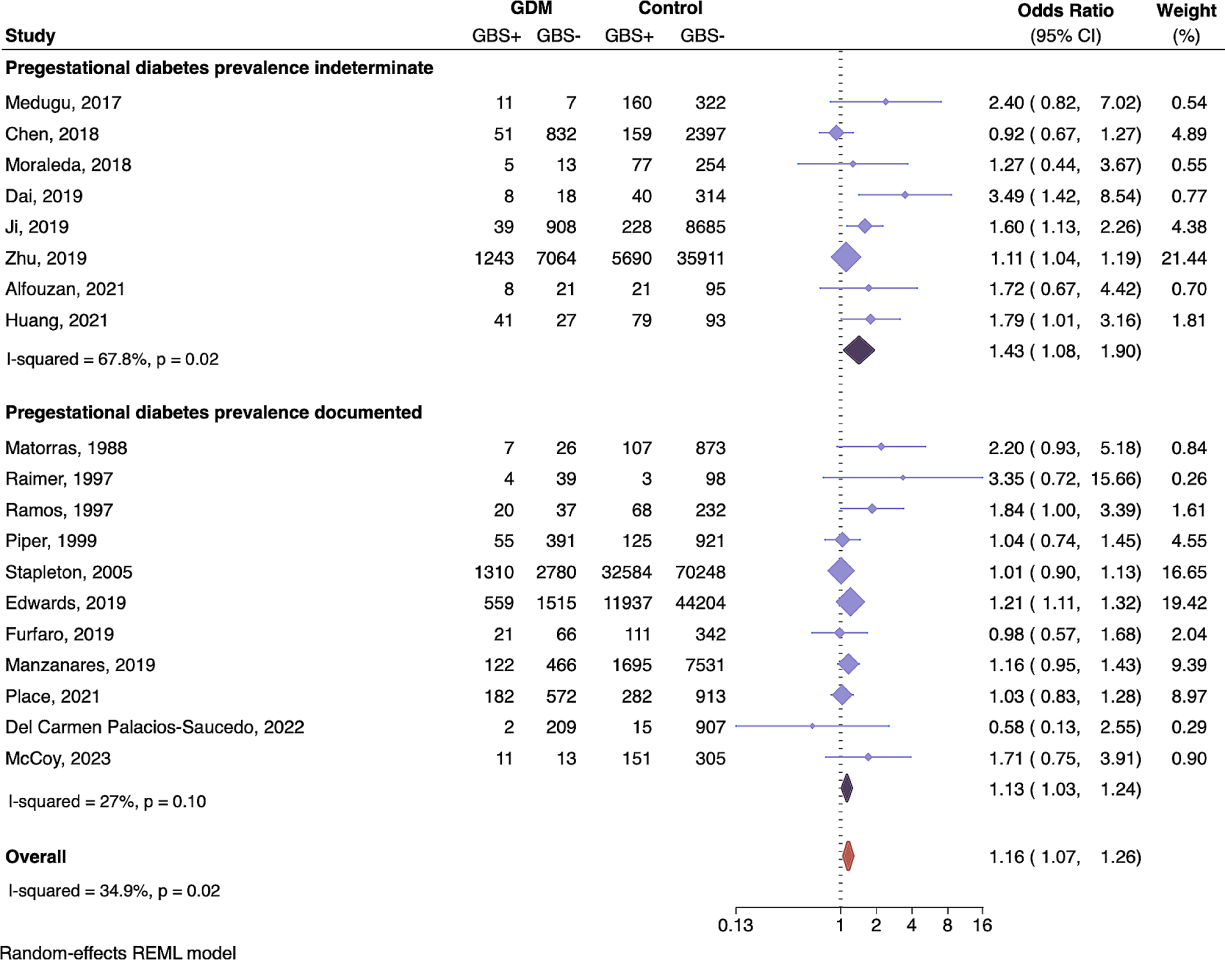
Association of gestational diabetes and GBS rectovaginal colonization. Forest plot of the association between gestational diabetes and GBS rectovaginal colonization presented as Odds Ratios (OR) for each study and respective 95% confidence intervals (CI). Studies are grouped by those that did not document (top) or did document (bottom) the prevalence of pregestational diabetes in their study population. The number of women with (GBS+) and without (GBS-) rectovaginal GBS carriage are presented for each study. The dotted black line demarcates no effect (OR = 1). The OR of individual studies are represented by light purple diamonds with shape size corresponding to the weight of the study as determined by random-effects modeling, and the paired horizontal lines indicate the 95% CI. Pooled ORs for each group are shown by the dark purple diamonds and the orange symbol represents the OR for all studies

A meta-analysis of the association between gestational diabetes and maternal rectovaginal GBS carriage revealed that women with GDM are 16% more likely to be colonized by GBS compared to women without GDM (pooled OR 1.16, CI 1.07–1.26, *P* = 0.003) (Fig. 2). Heterogeneity of all studies was moderate (I^2^ = 34.9, *P* = 0.02). A significant driver of heterogeneity was whether the prevalence of pregestational diabetes was accounted for in the study population; sub-analysis revealed that when pregestational diabetes prevalence was not documented, and possibly present in the GDM group, women with GDM had a 43% increased risk of GBS colonization (OR 1.43, CI 1.08–1.9, *P* = 0.01). Study heterogeneity was significantly greater among this subset of studies (I^2^ = 67.8, *P* = 0.02). When pregestational diabetes prevalence is accounted for (thus reliably excluded from the GDM group), study heterogeneity is mitigated (I^2^ = 27, *P* = 0.10), and women with GDM have a 13% increased risk of GBS colonization compared to the non-diabetic control group (OR 1.13, CI 1.03–1.24, *P* = 0.01) (Fig. 2). Per the GRADE [25, 42] approach, the overall quality of the evidence is moderate to low based on study limitations, publication bias, heterogeneity across studies, imprecision and indirectness.

### Association between pregestational diabetes and maternal GBS colonization

**Fig. 3:**
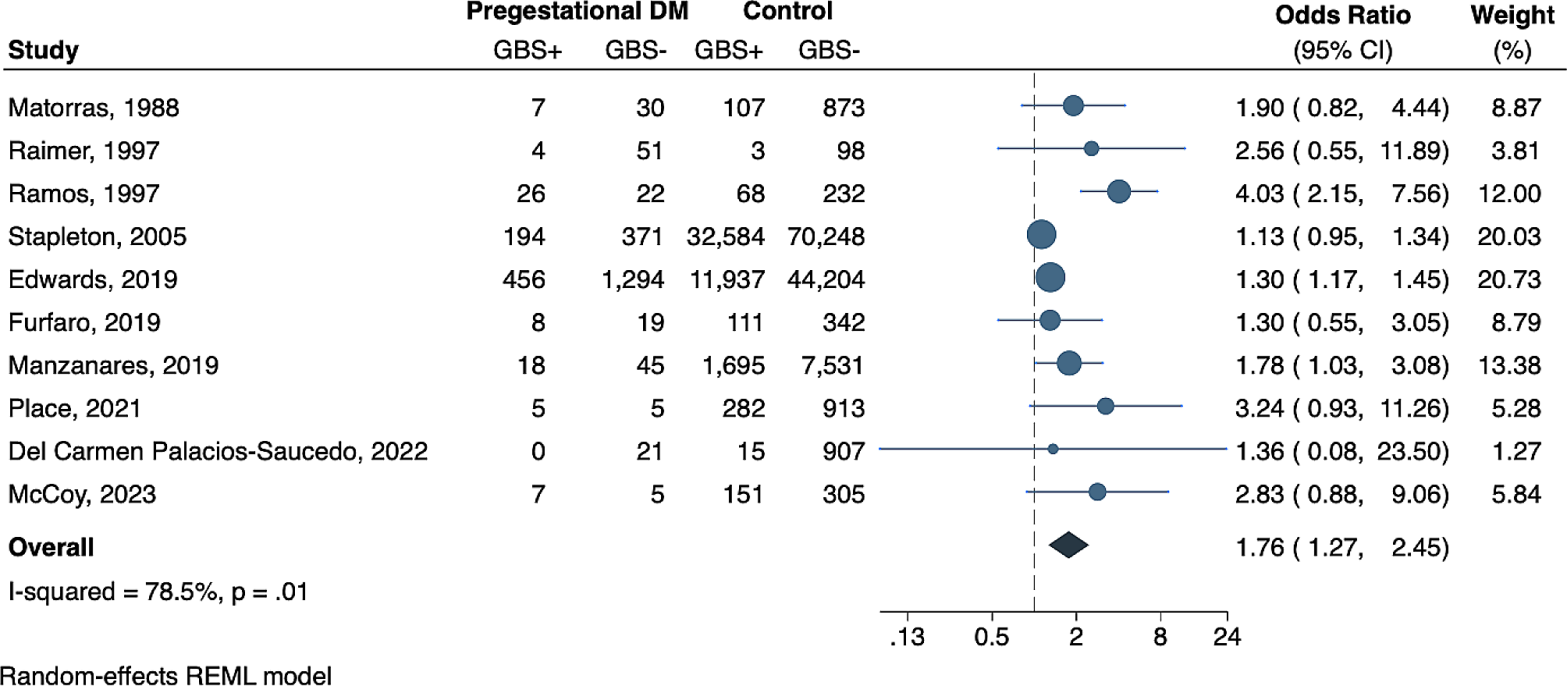
Association of pregestational diabetes and GBS rectovaginal colonization. Forest plot of the association between pregestational diabetes and GBS rectovaginal colonization presented as Odds Ratios (OR) for each study and respective 95% confidence intervals (CI). The number of women with (GBS+) and without (GBS-) rectovaginal GBS carriage are presented for each study. The dotted black line demarcates no effect (OR = 1). The OR of individual studies are represented by blue circles with shape size corresponding to the weight of the study as determined by random-effects modeling, and the paired horizontal lines indicate the 95% CI. Pooled ORs for each group are shown by the dark blue diamond

**Fig. 4:**
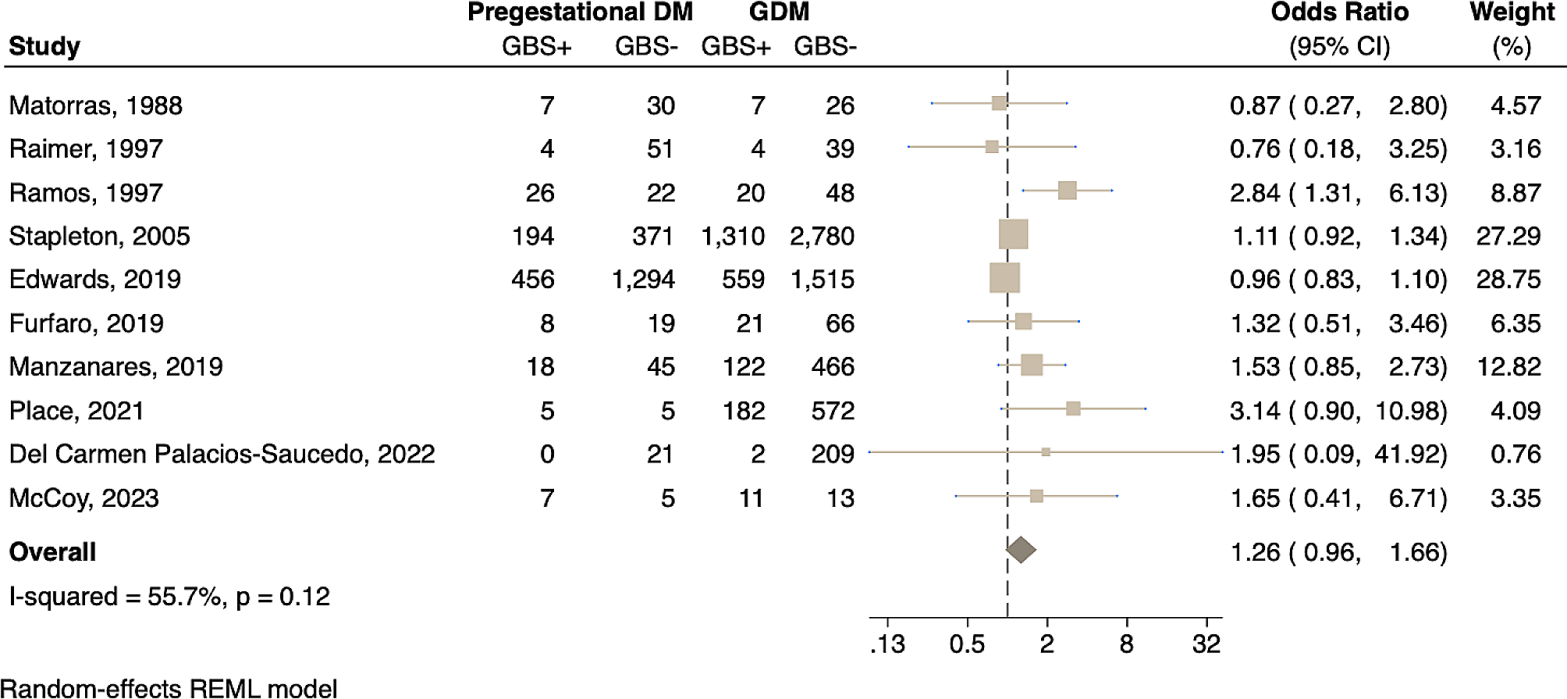
Comparison of diabetes types and associations with GBS rectobaginal colonization. Forest plot of the association between pregestational diabetes and GBS rectovaginal colonization relative to women with gestational diabetes, presented as Odds Ratios (OR) for each study and respective 95% confidence intervals (CI). The number of women with (GBS+) and without (GBS-) rectovaginal GBS carriage are presented for each study. The dotted black line demarcates no effect (OR = 1). The OR of individual studies are represented by squares with shape size corresponding to the weight of the study as determined by random-effects modeling, and the paired horizontal lines indicate the 95% CI. Pooled ORs for each group are shown by the dark beige symbol

We performed an additional sub-analysis to determine the independent association between pregestational diabetes and maternal GBS carriage, which revealed that women with pregestational diabetes have a 76% increased risk of rectovaginal GBS carriage compared to non-diabetic women (Fig. 3) (pooled OR 1.76, CI 1.27–2.45, *P* = 0.0008). There was a high degree of heterogeneity between studies (I^2^ = 78.5%, *P* = 0.01). Appreciating distinct pathophysiology and outcomes for women with pregestational diabetes vs. gestational diabetes, we assessed differences in risk of GBS colonization. There was no significant difference in risk of GBS rectovaginal colonization based on diabetes type (Fig. 4); women with pregestational diabetes had a similar risk of GBS rectovaginal colonization compared to those with gestational diabetes (pooled OR 1.26, CI 0.96–1.66, *P* = 0.09). Even so, it is possible that differences will resolve with a larger sample size.

### Publication bias and sensitivity analysis

**Fig. 5:**
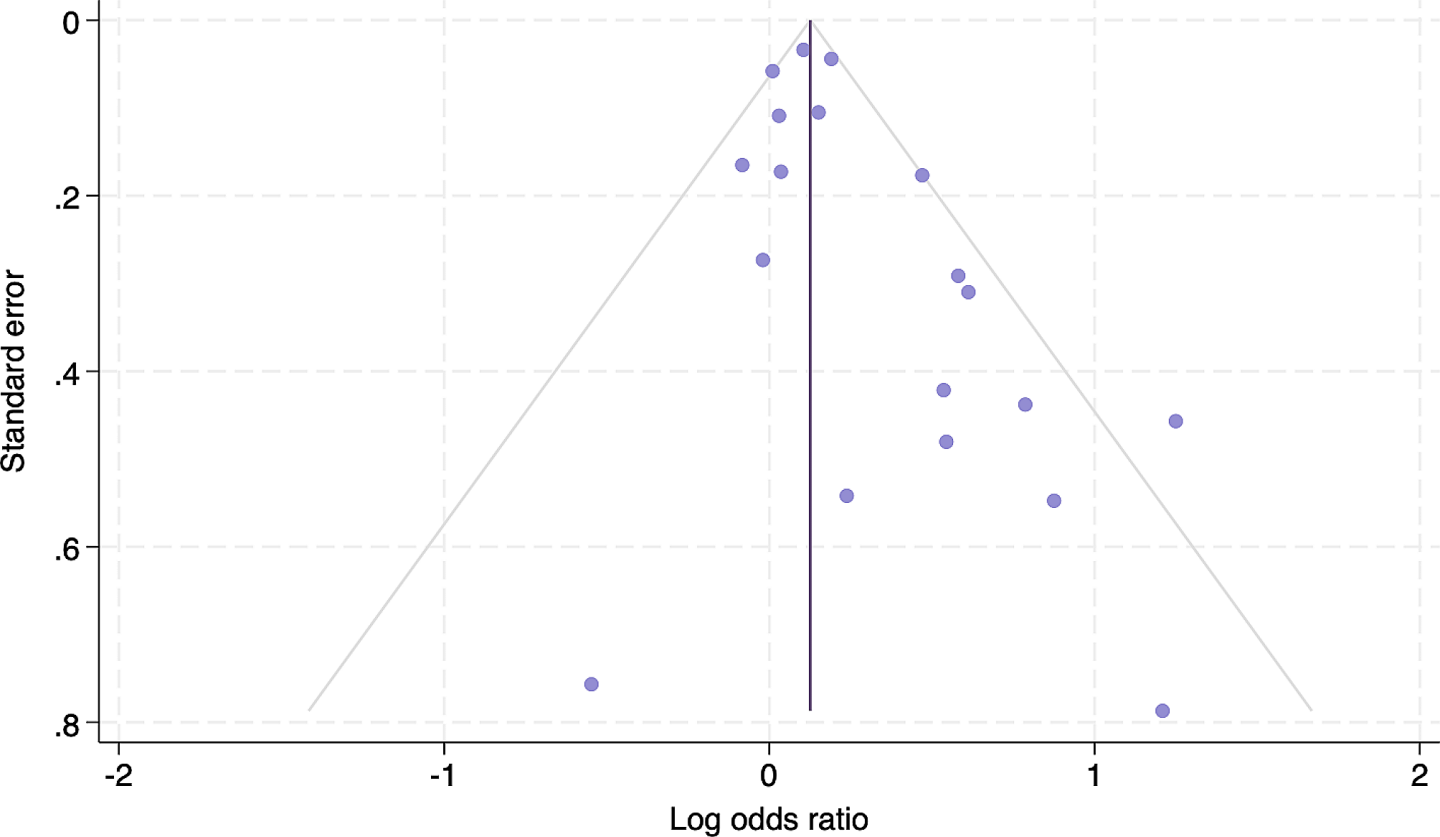
Risk of bias of included studies. Funnel plot for visual assessment of publication bias for all included studies. Circles represent individual study estimates (log odds ratio) against the respective standard error. The purple vertical line indicates the pooled OR. The gray lines mark the bounds of a pseudo 95% confidence interval

Visual assessment of the funnel plot (Fig. 5) shows asymmetrical distribution of studies, with publication bias confirmed by Egger’s test (*P* = 0.005). Sensitivity analysis included complete exclusion of studies that did not document or control for pregestational diabetic prevalence and exclusion of studies with high risk of bias. A meta-analysis was then performed on the remaining 8 studies (Raimer, Ramos, Stapleton, Edwards, Furfaro, Manzanares, Place, McCoy). Findings were robust; gestational diabetes was still associated with a 13% increased risk of rectovaginal GBS colonization (OR 1.13, 95% CI 1.02–1.25, *P* = 0.02), without significant shifts in study heterogeneity (I^2^ = 35.4%, *P* = 0.08).

## Discussion

To our knowledge, this is the first systematic review and meta-analysis examining the association between gestational diabetes mellitus and group B streptococcal rectovaginal colonization. We also performed subgroup analyses to assess independent effects of pregestational vs. gestational diabetes on risk of maternal GBS carriage. Our meta-analysis demonstrates that women with GDM have a significant 16% greater risk of being colonized by GBS, which may in part explain the increased risk of sepsis for neonates born to mothers with GDM [13, 14]. Sub-analysis revealed that women with pregestational diabetes have a 76% increased risk. However, our sub-analysis did not detect significant differences in risk based on diabetes type, consistent with reports from smaller cohort studies [17, 18]. Nevertheless, it is possible that women with pregestational diabetes have a heightened risk that requires a larger sample size to detect. We suspect that GBS risk is likely associated with the degree and extent of diabetic disease; we hypothesize that chronic disease (Type 1 and 2 DM) confers greater GBS risk than a comparatively transient disease course (GDM), but this requires further study.

GDM-mediated perturbations to critical host defenses such as immunity and the vaginal microbiota may mechanistically contribute to increased susceptibility to GBS carriage. GDM leads to altered neutrophil, NK, T cell and macrophage abundance and/or activity both in the peripheral blood and at the maternal-fetal interface [10, 43–46]. While the direct role of the vaginal microbiome in propagating or limiting GBS colonization remains largely unknown, it is well-appreciated that members of the vaginal microbiota play direct and indirect roles in maintaining reproductive health and pregnancy outcomes and *Lactobacillus* dominance is considered a hallmark of an optimal vaginal community [47–51]. During pregnancy, the stability of the vaginal microbiota increases with *Lactobacillus* enrichment and overall lower alpha diversity [52, 53]. In non-diabetic pregnancy, non-*Lactobacillus* dominance or dominance by *Lactobacillus gasseri* has been associated with increased risk of GBS colonization [41, 54]. GDM disrupts the vaginal microbiota with increased diversity and enrichment of nonoptimal members including *Bacteroides*, *Klebsiella*, *Enterococcus*, and *Enterobacter* and *Staphylococcus* [11, 12, 55], of which *Staphylococcus* has been positively associated with vaginal GBS colonization [56]. Microbial communities inherited by neonates are also impacted by GDM reflected by increased colonization by *Streptococcaceae* and *Enterococcaceae* which may contribute to worse neonatal outcomes upon GBS encounter [11, 57, 58]. Indeed, in a preclinical model of GDM, we recently showed that GDM susceptibility to fetoplacental infection was driven by perturbations to maternal immunity and vaginal microbial communities in addition to pathogenic bacterial adaptations [59].

The heterogeneity observed in the 19 studies representing 10 different countries greatly enhance the generalizability of our findings and were in part explained by the presence of pregestational diabetes as a potential confounder, as revealed by sub-analyses. Other possible drivers of heterogeneity are differences in study populations including: sample size, severity of diabetes, discrepancies in access to prenatal care thereby impacting who was included in hospital or clinic-based studies, differential presence of confounding metabolic disease such as obesity, variation in inclusive representation of underrepresented or under-resourced communities, and geographical variation in GBS prevalence [1]. Other notable sources of heterogeneity between studies includes regional variations in healthcare utilization due to differences in healthcare coverage (e.g. universal vs. private) and access (e.g. transportation, clinic schedule) to different healthcare systems. Communities with ample providers and institutions with open access will best capture inclusive estimates of GBS risk in the diabetic population, whereas communities with relatively inaccessible healthcare will selectively sample women with more resources. We accounted for potential selection bias by considering whether study populations were representative of the general population with inclusion of women across various socioeconomic and racial/ethnic groups.

There was also variability in GBS screening and IAP practices across studies, where about half of the studies were conducted in settings that had no established guidelines for screening or prophylactic treatment, while other studies occurred in settings with risk-based or universal screening. The lack of infrastructure and protocol for routine screening may impact detection and treatment rates in clinical practice. We suspect that such limitations were mitigated by study design in studies where GBS colonization rates were a primary measure. Differences in GBS sampling (vaginal vs. rectovaginal vs. vaginoperineal) and laboratory identification techniques can also affect detection and lead to underestimation of GBS colonization rates. In our assessment, studies that did not adhere to CDC standards for GBS sampling (rectovaginal) and laboratory detection were assigned a higher score for bias risk.

Differences in severity of diabetes may also explain variation between studies; a recent study found that pregnant women with better glycemic control (Hemoglobin A1c < 6.5%) had a 45% decreased risk of GBS rectovaginal colonization [60]. However, a few studies report contrasting evidence; two reports found no differences in GBS colonization status for pregnant women requiring insulin therapy [18, 61], and another study found no differences in GBS status between pregnant diabetic women requiring greater than 20 U insulin therapy vs. those requiring less than 20 U per day [62]. Regional differences in affordability and access to medications and healthcare providers likely also contribute to differences in diabetic severity and variability among studies. The association between maternal GBS colonization and diabetes severity as indicated by glycemic control and medical management (insulin treatment vs. lifestyle modification) requires further study.

Notable limitations of some of the studies incorporated in this analysis include a lack of information about GDM or pregestational diabetic severity (e.g. HbA1C or need for medical intervention), and variable or unstated GDM diagnostic criteria. The two most commonly utilized GDM screening approaches vary in sensitivity; one-step screening (a single 75 gram glucose challenge during fasting) has a higher rate of positive screening and thus GDM diagnosis compared to the two-step screening approach (50 gram glucose challenge in non-fasting state and if positive a second 100 gram glucose challenge during fasting) [63, 64]. As such, an important source of variability among included studies is difference in GDM diagnostic sensitivity. It is possible that some studies underestimated the presence of glucose intolerance in their population and missed potential GDM diagnoses. Consequently, the risk of GBS colonization may be underestimated. The differences in GDM diagnostic approaches should be considered in future studies. Although we did not account for one-step vs. two-step approaches in our evaluation of study bias and quality, we did consider the source of diagnosis; studies in which GDM diagnosis was not described or self-reported had a higher bias score compared to diagnoses reported by medical professionals and with criteria specified.

The presence of publication bias is another limitation and may mean that the risk is overestimated as studies with negative findings are less likely to be published. Nevertheless, the association between GDM and GBS carriage remains when high-risk and potentially confounded studies are excluded. Thus, we are confident that our findings withstand the observed limitations.

## Conclusions

Ultimately, this systematic review and meta-analysis of 19 studies representing over 260,000 women across the globe revealed gestational diabetes as a novel risk factor for maternal rectovaginal colonization by group B *Streptococcus*. Considering that up to 46% of neonates with GBS invasive disease are born to women with no currently recognized risk factors for GBS transmission, GDM may be an important risk that is not yet clinically recognized. A critical future direction is to assess neonatal infection rates in women with GDM and to determine if effective glucose management plays a role in limiting GBS morbidity and mortality in neonates. Ultimately, expanding our knowledge of additional risk factors for GBS neonatal transmission will improve strategies for screening, preventing, and treating fetal and neonatal GBS disease.

### Electronic supplementary material

Below is the link to the electronic supplementary material.

Supplementary Material 1

## Data Availability

The datasets supporting the conclusions of this article are included within the article, and in the cited studies included in the meta-analysis.

## Abbreviations

GBS: Group B Streptococcus
IAP: Intrapartum Antibiotic Prophylaxis
EOGBS: Early-onset GBS Disease
GDM: Gestational Diabetes Mellitus
