# Supplementary material for: Gestational diabetes as a risk factor for GBS maternal rectovaginal colonization: a systematic review and meta-analysis
Source: BMC Pregnancy Childbirth. 2024 Jul 20;24:488. doi: 10.1186/s12884-024-06694-7 (PMC11264770; doi:10.1186/s12884-024-06694-7)
Supplement: Supplementary file 1 — Supplementary Material 1 [file 12884_2024_6694_MOESM1_ESM.pdf]

Gestational diabetes as a risk factor for GBS maternal rectovaginal colonization: a systematic review and meta-analysis

Vicki Mercado-Evans, Jacob J. Zulk, Zainab A. Hameed, and Kathryn A. Patras

SUPPLEMENTAL MATERIALS

Supplemental table 1: Search terms utilized.

Database	Search terms
EMBASE	(('gestational diabetes'/exp OR 'gestational diabetes' OR (gestational AND ('diabetes'/exp OR diabetes))) AND ('group b streptococcus'/exp OR 'group b streptococcus' OR (('group'/exp OR group) AND b AND ('streptococcus'/exp OR streptococcus))) OR gbs OR 'streptococcus agalactiae'/exp OR 'streptococcus agalactiae' OR (('streptococcus'/exp OR streptococcus) AND agalactiae) OR ('chorioamnionitis'/exp AND 'group b streptococcus') OR ('newborn sepsis'/exp AND 'group b streptococcus') OR ('newborn meningitis'/exp AND 'group b streptococcus') OR ('newborn'/exp AND cocci AND 'sterile site') OR agalactiae) AND [english]/lim AND ([article]/lim OR [article in press]/lim OR [conference paper]/lim OR [data papers]/lim OR [letter]/lim OR [note]/lim OR [preprint]/lim) AND [humans]/lim NOT 'guillain barre syndrome' NOT 'in vitro' NOT 'ex vivo' NOT 'animal/exp' NOT 'cells' NOT 'tilapia' NOT 'zebra fish' NOT 'bovine' NOT 'breast milk' NOT 'fish' NOT 'phylogeny/exp' NOT 'case study' NOT 'case report' NOT 'urinary tract infection/exp' NOT 'non-pregnant' NOT 'endocarditis' NOT 'murine' NOT 'mouse' NOT 'primate':ab,ti
PubMed	“Gestational diabetes and group b streptococcus”

Supplemental table 2: Studies excluded upon full-text appraisal.

Rationale for study exclusion	Studies excluded
Did not distinguish diabetes type *n* = 22	Arias et al., 2022, Akhlaghi et al., 2007, Bey et al., 1992, Chen et al., 2019, Chen et al., 2023, Embil et al. 1978, Ernest et al., 2015, Gopal Rao et al., 2019, Herrera et al., 2023, Horvath et al., 2013, Horvath et al., 2013, Kessous et al., 2012, Lukic et al., 2017, Moghaddam et al., 2010, Mulla et al., 2012, Musleh et al., 2018, Najmi et al., 2013, Obata-Yasuoka, 2012, Rick et al., 2017, Shah et al., 2011, Strus et al., 2009, Zhang, 2023
Did not assess GDM as a risk factor *n* = 18	Akman et al., 2001, Alzuheiri et al., 2021, Belhadi et al., 2020, Berikopoulou et al., 2021, Botelho et al., 2018, Chaudhary et al., 2015, Dadi et al., 2022, Dahl et al., 2003, Elvedi-Gašparović et al., 2008, Ji et al., 2017, Jones et al., 2006, Khamching et al., 2017, Khan et al., 2015, Nahaei et al., 2007, Philipson et al. 1996, Pykało-Gawińska et al., 2021, Tor-udom et al., 2006, Werawatakul et al., 2001
Incomplete information *n* = 3	Clouse et al., 2019, Regan et al., 1991, Siqueira et al., 2019
Exclusion and inclusion criteria not reported *n* = 1	Hammoud et al., 2002
Article not in English *n* = 1	Jerbi et al., 2007
Article not accessible online *n* = 1	Prośniewska, M. et al., 2014

Supplemental table 3: Risk of bias and quality assessment scale.

**SELECTION BIAS**	
**Representativeness of the exposed group**	Score & Comments
0- Representative of the general population	
1- Moderately limited in its representation of the general population	
2- Only representative of a select population	
3- No description of the sampling strategy (inclusion/exclusion criteria)	
**Representativeness of the control group**	Score & Comments
0- Representative of the general population	
1- Moderately limited in its representation of the general population	
2- Only representative of a select population	
3- No description of the sampling strategy (inclusion/exclusion criteria)	
**Sample size**	Score & Comments
0- Satisfactory or justified: power calculations reported or $n \geq 488$	
1- Not satisfactory or justified	
**COMPARABILITY**	
**Comparability of the cohorts**	Score & Comments
0- Exposed and non-exposed matched in design or confounders adjusted for in analysis	
1- Cohorts are not matched and confounders not adjusted for in analyses, or information not provided	
**MEASUREMENT BIAS**	
**Ascertainment of group B Streptococcal carriage**	Score & Comments
0- Sampling, culturing and identification thoroughly performed and described with substantial adherence to CDC guidelines	
1- Sampling, culturing and identification thoroughly performed and described but without substantial adherence to CDC guidelines	
2- Limited or no description	
**Ascertainment of GDM exposure**	Score & Comments
0- Diagnosis reported from medical professional or medical records with diagnostic criteria specified	
1- Diagnosis reported from medical professional or medical records with diagnostic criteria not specified	
2- Self-reporting/questionnaires	
3- No description	
**POTENTIAL OF BIAS FROM OTHER METABOLIC DISEASES**	
**Presence of other metabolic disease(s) in cohort**	Score & Comments

0- Prevalence of obesity and pregestational diabetes in exposed and nonexposed groups is provided, or other metabolic diseases were part of exclusion criteria	
1- Prevalence of obesity and pregestational diabetes not provided or not part of exclusion criteria	

Supplemental table 4: Quality and risk of bias scores for each study.

Study	Selection bias	Cohort Comparability	Measurement bias	Bias from other metabolic diseases	Total Score	Overall Bias
Matorras, 1988	6	1	0.5	1	8.5	High
Raimer, 1997	2.5	1	1	1	5.5	Mod.[a]
Ramos, 1997	2	0	1	0	3	Low
Piper et al., 1999	4	1	0.5	1	6.5	High
Stapleton et al., 2005	0	0	2.5	0	2.5	Low
Medugu et al., 2017	0	1	1.5	1	3.5	Mod.
Chen et al., 2018	0	0	1	1	2	Low
Moraleda et al., 2018	1	1	3.5	1	6.5	High
Dai et al., 2019	3	1	2	1	7	High
Edwards et al., 2019	0	0	2	1	3	Low
Furfaro et al., 2019	0	0	1	0	1	Low
Ji et al., 2019	2	1	1.5	1	5.5	Mod.
Manzanares et al., 2019	2	0	3	0	5	Mod.
Zhu et al., 2019	0	0	3.5	1	4.5	Mod.
Alfouzan et al., 2021	3	0	2	1	6	Mod.
Huang et al., 2021	3	0	2	1	6	Mod.
Place et al., 2021	4	0	0.5	1	5.5	Mod.
Del Carmen Palacios-Saucedo, et al, 2022	4	1	3	0.5	8.5	High

McCoy et al., 2023	2	0	1	0	3	Low
Total possible points for category	*7*	*1*	*5*	*1*	*14*	-

[a]Mod. = moderate
